# How patients with gout become engaged in disease management: a constructivist grounded theory study

**DOI:** 10.1186/s13075-018-1608-x

**Published:** 2018-06-01

**Authors:** Alyssa Howren, Susan M. Cox, Kam Shojania, Sharan K. Rai, Hyon K. Choi, Mary A. De Vera

**Affiliations:** 1 Faculty of Pharmaceutical Sciences, University of British Columbia, 2405 Wesbrook Mall, Vancouver, BC V6T 1Z3 Canada; 2 Arthritis Research Canada, Richmond, BC Canada; 3 Collaboration for Outcomes Research and Evaluation, Vancouver, BC Canada; 4 University of British Columbia, School of Population & Public Health, Vancouver, BC Canada; 5 Faculty of Medicine, Department of Medicine, Division of Rheumatology, University of British Columbia, Vancouver, BC Canada; 6 Department of Nutrition, Harvard T.H. Chan School of Public Health, Boston, MA USA; 7 Population Health Sciences Program, Graduate School of Arts and Sciences, Harvard University, Cambridge, MA USA; 8 Division of Rheumatology, Allergy and Immunology, Department of Medicine, Massachusetts General Hospital, Harvard Medical School, Boston, MA USA

**Keywords:** Gout, Qualitative research, Grounded theory, Disease management

## Abstract

**Background:**

Prior qualitative research on gout has focused primarily on barriers to disease management. Our objective was to use patients’ perspectives to construct an explanatory framework to understand how patients become engaged in the management of their gout.

**Methods:**

We recruited a sample of individuals with gout who were participating in a proof-of-concept study of an eHealth-supported collaborative care model for gout involving rheumatology, pharmacy, and dietetics. Semistructured interviews were used. We analyzed transcripts using principles of constructivist grounded theory involving initial coding, focused coding and categorizing, and theoretical coding.

**Results:**

Twelve participants with gout (ten males, two females; mean age, 66.5 ± 13.3 years) were interviewed. The analysis resulted in the construction of three themes as well as a framework describing the dynamically linked themes on (1) processing the diagnosis and management of gout, (2) supporting management of gout, and (3) interfering with management of gout. In this framework, patients with gout transition between each theme in the process of becoming engaged in the management of their gout and may represent potential opportunities for healthcare intervention.

**Conclusions:**

Findings derived from this study show that becoming engaged in gout management is a dynamic process whereby patients with gout experience factors that interfere with gout management, process their disease and its management, and develop the practical and perceptual skills necessary to manage their gout. By understanding this process, healthcare providers can identify points to adapt care delivery and thereby improve health outcomes.

## Background

Despite the availability of effective medication therapy in the form of urate-lowering therapy (ULT), studies have consistently reported suboptimal outcomes, including repeated flares [1], increased cardiovascular mortality [2], and excess all-cause mortality [2, 3], for individuals with gout, the most common inflammatory arthritis in men [4]. Factors contributing to suboptimal patient outcomes include poor adherence to ULT, with rates ranging from 10% to 46% [5], and insufficient quality of care [6–8]. As such, efforts are presently focused on optimizing care delivery and improving outcomes for patients with gout [9, 10], including models of care delivery involving allied healthcare providers such as rheumatology nurses [11] and pharmacists [12, 13].

Aside from novel models of care, also important to improving the quality of care for gout is an understanding of the patient’s perspective, particularly through applying qualitative inquiry because this has the capacity to elucidate the discordance between evidence-based practice and the reality of managing gout [14]. Qualitative research in gout has been published in the United States, the United Kingdom, Australia, New Zealand, and the Netherlands, with a recent thematic synthesis by our group showing that studies have primarily reported barriers to optimal management of gout from patients’ as well as providers’ perspectives, primarily situated within traditional care delivery models [15]. Although a 2014 study evaluated factors that influence ULT adherence [16] and a 2017 study explored solutions for self-management among African American male veterans [17], the findings are limited in scope with respect to a focus on medication use [16] and a distinct patient sample [17]. Current knowledge gaps include how patients with gout can best be supported in the context of receiving care. As such, to inform optimal care delivery through a patient-centered lens, we aimed to explore individual experiences with gout to understand how they become engaged in the management of gout in the context of receiving care.

## Methods

### Study design

We conducted a qualitative study nested within the Virtual Gout Study, a longitudinal proof-of-concept study evaluating an eHealth-supported collaborative care model involving rheumatology, pharmacy, and dietetics for gout in British Columbia, Canada [18]. In brief, in this novel decentralized model, eight community rheumatologists’ electronic medical records (EMR) for consented participants with gout were shared with a study pharmacist and study dietitian who provided consultations, respectively, via telephone. As such, this shared EMR supported remote communication and collaboration among health professionals. The descriptive qualitative study was informed by constructivist grounded theory, an approach that is well suited to the study of social processes and gaining an in-depth understanding of participants’ lived experiences [19, 20].

### Participant recruitment

We invited individuals from the Virtual Gout Study, which included patients with confirmed gout who were seen in one of four participating rheumatology practices and had at least one flare in the past year and serum uric acid (SUA) level > 360 μmol/L in the past 2 months (at time of recruitment) to participate in our qualitative study. According to the Virtual Gout Study protocol, participants (1) were seen by their rheumatologists on an as-needed basis; (2) had monthly (or as-needed) telephone consults with the study pharmacist, including medication reviews (e.g., discussion of ULT dosage, medication adherence, discontinuation of unnecessary medications), and discussion of laboratory test results; and (3) one telephone consult with the study dietitian regarding dietary recommendations for gout. To explore a range of experiences, we purposefully sampled interview participants according to SUA level and self-reported adherence using the five-item version of the Compliance Questionnaire for Rheumatology (CQR5) [21–24], as measured in the Virtual Gout Study. We applied the criteria of completion of a minimum of 6 months of follow-up in the Virtual Gout Study with at least one pharmacist and one dietitian consult, able to provide informed consent, having access to a phone, and able to comprehend and speak English.

### Data gathering

Semistructured interviews, using adaptable probes and prompts, were conducted with participants by a single author (AH) over the telephone. Each interview was started by briefing the participant on the subject matter and purpose and situating the participant as the expert early in the interview [25, 26]. A topic guide with open-ended questions was developed and revised by study authors (AH, SMC, SKR, MADV), and the interview was focused on exploring participants’ experiences with gout before and during the Virtual Gout Study, management of gout, perceptions of disease activity, and beliefs and behaviors surrounding gout medications. Interviews were recorded using a WS-853 digital voice recorder (Olympus, Center Valley, PA, USA). Professional transcription service providers transcribed each audio-recorded interview.

### Analysis

We followed three main steps of the coding process of constructivist grounded theory: initial coding, focused coding and categorizing, and theoretical coding [19]. For the initial coding phase, we conducted line-by-line coding. Focused coding narrowed the scope of the qualitative analysis by identifying initial codes that held analytical significance or were repetitive. Last, theoretical coding was done with the aim of interpreting relationships between constructed categories [19]. On the basis of emerging analysis as well as prior knowledge that poor ULT adherence [5] and management [6–8] underlie suboptimal health outcomes in gout, we explored previous analytic constructs that pertain to treatment adherence to inform the emerging theoretical codes [27]. Analytical techniques such as the constant comparative method and memo-writing were applied throughout [19, 28]. Data gathering and analysis were carried out in an iterative process such that participants were interviewed until saturation was achieved. This is the point where no new insights into the constructed categories and themes emerged [29]. We used NVivo 11 (QSR International, Doncaster, Australia) for all analyses. This study was reviewed and approved by the University of British Columbia Behavioural Research Ethics Board (H16–02061).

## Results

Twelve participants with gout (ten males, two females; mean age, 66.5 ± 13.3 years) were interviewed over the telephone. Mean SUA as recorded in the Virtual Gout Study nearest the time of interview was 387 μmol/L (± 110 μmol/L). Six participants had SUA > 360 μmol/L and/or were classified as nonadherent by the CQR5. All participants were prescribed ULT at the time of the interview. The average duration of the interviews was 33 minutes. The analysis resulted in the construction of three themes: (1) processing the diagnosis and management of gout, (2) supporting management of gout, and (3) interfering with management of gout. In addition, we used an explanatory framework to illustrate the process of becoming engaged in gout management.

### Themes

#### Theme 1: processing the diagnosis and management of gout

The first theme, processing the diagnosis and management of gout, which encompassed how participants learn to navigate their diagnosis, comprised conceptual categories of: (1) adapting to gout, (2) searching for reason, and (3) testing the waters (Table 1). *Adapting to gout* describes how participants found ways to modify their lifestyles, including practical changes, acclimatizing to the pain, and modifying diet. Practical changes included participants adjusting their activity levels on the basis of disease activity and making accommodations (e.g., footwear, aids/devices), whereas dietary modifications included identifying and avoiding personal triggers such as acidic foods, alcohol, and seafood. *Searching for reason* describes the process shared by some participants in which they sought to find reasons for having gout, such as questioning the relationship between diet and a high SUA or undergoing the emotional experience of questioning why they have gout and why they have to endure such pain. Last, *testing the waters* is a process in which participants mentioned instances when they trialed their diet or modified their medications. This self-experimentation often occurred during a period when participants reached a level of comfort with gout management or an asymptomatic period. For one participant, concern about side effects of gout medications preceded modification to medications.

**Table 1:** Conceptual categories and example quotations from participants for theme 1

Theme 1: Processing the diagnosis and management of gout	
Conceptual category	Example quotations
Adapting to gout	*“I’m very, I’m very careful for what I am eating or, or drinking.”* (Participant 5, male) *“You plan your day around how you feel.”* (Participant 11, male)
Searching for reason	*“I don’t know whether it was because I was particularly dehydrated when I took the blood test or maybe I’d consumed more of the triggers leading up to it.”* (Participant 8, male) *“If I have a, a gout what’s this, a flared up, I always have tears in my eyes, why me, why me, I ask myself, why me.”* (Participant 5, male)
Testing the waters	*“Because I hadn’t been having flare ups, I, I felt I could indulge a little bit more in some of the foods that I knew that were triggers.”* (Participant 8, male) *“So I took it [allopurinol] every other day for a while and I held my own and then I tried every second day for maybe a couple of weeks.”* (Participant 7, female)

#### Theme 2: supporting management of gout

The second theme of *supporting management of gout* comprised six conceptual categories: (1) being organized, (2) identifying motivation, (3) taking control, (4) seeing a difference, (5) resonating importance of gout medications, and (6) developing acceptance. A common supporter of managing gout that participants identified was a sense of *being organized*, whether an inherent or acquired behavior. Some participants were taking several medications for other conditions, and therefore an emphasis was placed on the necessity of taking and scheduling their treatments. Many participants discussed how taking their gout medications had become a routine integrated into their daily schedule or was paired with an already established daily activity.

The category *identifying motivation* describes the reason why participants are compelled to take their gout medications. Most participants stated that they continued to take their medications to avoid the immense pain experienced during gout flares. As such, it seems that most participants had made the connection between adhering to daily ULT and the prevention of future pain from gout. A few participants mentioned the need to get back to day-to-day activities to improve their health as a significant motivator as well as to avoid visits to the hospital or their physician’s office.

*Taking control* refers to participants having an active role in managing their gout. Participants relayed a sense of personal responsibility such as being proactive and taking initiative, acknowledging the importance of knowing one’s own body (e.g., triggers of gout flares), and feeling that “*my health is my concern*” (*participant 5, male*). Also mentioned by participants was being proactive in terms of searching for information about gout online and requesting an appointment with a specialist. In addition, some participants mentioned having a personal plan to deal with future gout flares, including knowing when to take colchicine, which appeared to establish confidence in managing their disease.

The category *seeing a difference* refers to moments during treatment in which participants realized the role that medications and diet play in modifying their gout symptoms, such as when stopping or initiating gout medications and then noticing a change in disease activity. The process of altering ULT or diet and observing a reaction describes a self-initiated learning experience for participants. From another perspective, a participant with high SUA or gout flares noticed the reduction of symptoms after starting allopurinol: “*There was a drastic improvement after 6 months and then gradual improvements ever since*” (*participant 1, male*).

Related to this is the category *resonating importance of gout medications*, which details how participants attribute the improvement in their gout symptoms as a direct result of their gout medications. Consequently, the majority of participants expressed being committed to taking their medication and shared the common sentiment of “*I won’t stop taking those medicines*” (*participant 5, male*).

Several participants with gout remarked on *developing acceptance* in terms of medications and the prognosis of gout. Developing acceptance describes the hurdles overcome by participants toward being in a position to actively manage their gout. Some participants discussed the acceptance of medications such as accepting the side effects and the longevity of ULT. This encompasses knowing the potential side effects and ultimately deciding that the benefits of medication outweigh the potential for adverse reactions. Although a general resistance to taking medications also seems to be involved in this process, as one participant reflected on his decision making, “*I don’t wanna take it, but I have no choice. I have to take it every day*” (*participant 5, male*). Additional example quotations pertaining to this theme are provided in Table 2.

**Table 2:** Conceptual categories and example quotations from participants for themes 2 and 3

Theme 2: Supporting management of gout	
Conceptual category	Example quotations
Being organized	*“Well, I’m on other medications, so I’ve got a very regimented schedule when I take a medication.”* (Participant 1, male) *“It’s like brushing my teeth now, I gotta do it.”* (Participant 2, male)
Identifying motivation	*“If I don’t take my medication, I don’t want to get sick, right, because I’ve got to take care of my family and my husband and my housework too… and then I do my volunteering too.”* (Participant 3, female) *“Remembering what it’s like to have difficulty getting, getting your shoe on and walking around*.” (Participant 9, male)
Taking control	*“I mean the bottom line is I’m the patient and know my body so ultimately it becomes my responsibility.”* (Participant 12, male) *“then in my you know research online, I did a little bit more, I discovered a few more things and what the, what the causes were.”* (Participant 8, male)
Seeing a difference	*“Now it’s down to about 350, 360, which is obviously a huge difference taking the medication.”* (Participant 2, male) *“Well, it was about a year after but yeah, it (gout) came back, and I stopped it (medication) myself. I, I shouldn’t have. I probably should just have continued, you know.”* (Participant 6, male)
Resonating importance of gout medications	*“I really had the suspicion the way in which I’ve, I’ve reacted to the sole, solely to the medication change.”* (Participant 9, male) *“The lesson I’ve learnt is not to stop the allopurinol.”* (Participant 6, male)
Developing acceptance	*“[rheumatologist] said it’s probably taken me 30 years to get this bad so it’s not gonna go away in five minutes.”* (Participant 11, male) *“I mean you know like you wake up one day and you’ve got, got this funny pain in your body, you go to see the doctor and ultimately you go through the process.”* (Participant 12, male)
Theme 3: Interfering with management of gout	
Conceptual category	Example quotations
Disliking taking medication	*“I don’t particularly like relying on medication in general so I guess it’s just personal philosophy.”* (Participant 8, male) *“I ended up at you know taking more and more medications to the stage that it, I wasn’t really comfortable with that.”* (Participant 9, male)
Fearing side effects	*“And I guess not knowing, not knowing what the medication’s really gonna do say 10 years from now. Obviously they don’t make medications to kill you, right?”* (Participant 2, male) *“But then again you know the side effect and all that is I’m so scared.”* (Participant 3, female)
Affecting personal identity	*“You know like if I had, if I had any choice in the matter, I’d, I’d rather be in a position where you know like I’m, I have my health back that I had in my youth but that’s a dream.”* (Participant 12, male) *“Well if he says women don’t have gout, what’s this in my toes and why did they give me shots of whatever at the hospital and why did they extract what they told me was tophi.”* (Participant 7, female)
Forgetting medications	*“Not on purpose. I, I go away for a weekend for example and leave it at home, just because I’d forgotten it.”* (Participant 9, male) *“Well if I do, I just take it a little bit later, that’s all.”* (Participant 10, male)
Lacking knowledge/being misinformed	*“Well, I only took it periodically, maybe for a week and my gout rescinded. So I didn’t see any sense in taking it again.”* (Participant 1, male) *“Yeah, it’s, because you know just my understanding of, of my medications, I took it wrong.”* (Participant 5, male)

#### Theme 3: interfering with management of gout

The third theme, *interfering with management of gout*, describes challenges that participants with gout encounter. Three of the five conceptual categories, *disliking taking medication*, *fearing side effects*, and *affecting personal identity*, represent perceptual barriers, whereas the last two categories, *forgetting medications* and *lacking knowledge or being misinformed*, represent practical barriers (Table 2).

The conceptual category *disliking taking medications* captures a general aversion of some participants toward consuming medications. Many expressed feeling uncomfortable with taking medications, especially daily medications or a number of different medications, whereas another expressed how one can “*just have a mental block in your head about taking medications*” (*participant 2, male*).

Four participants expressed *fearing side effects* of their gout medications, such as how the medications could harm their kidneys.

*Affecting personal identity* describes a phenomenon in which some participants undergo a process of self-reflection and may feel reluctance to accepting their diagnosis. Indeed, a disposition toward taking daily ULT can be fueled by the reluctance to accept being diagnosed with a chronic disease. For some participants, this stems from having misconceptions about gout or knowledge of the misleading stereotypes associated with gout. For other participants, being diagnosed with a chronic condition prompted reflection on their own age and health status.

The final two categories identified practical barriers to optimal gout management, specifically *forgetting medications* and *lacking knowledge/being misinformed*. One participant shared how forgetting allopurinol was “*not on purpose*” (*participant 9, male*), and another described how, for him, “*It’s just not forgetting, it’s just ah, just being lazy*” (*participant 2, male*). For one participant, forgetting seemed to be connected to lacking knowledge, with the belief that ULT is “*built up after, you know, a week of taking it straight, missing it one day is probably not going to be detrimental, right?*” (*participant 2, male*). An additional frequent barrier voiced by participants was insufficient education about gout or the medications being prescribed. Narratives expressed by participants included the misconception of thinking there is no “cure” for gout, believing that ULT has a cumulative effect to prevent against future gout attacks, and misunderstanding medication directions. A common experience shared by participants was the decision to discontinue their ULT early because they seemed unconvinced of the need for daily medication and were unaware of the preventive nature of ULT.

#### Framework for understanding engagement in gout management

**Fig. 1:**
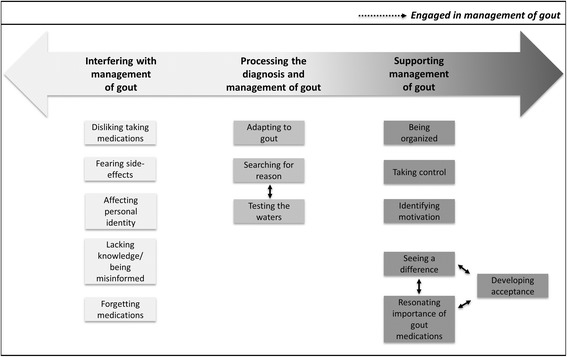
Schematic of three main themes constructed in the qualitative analysis to describe the process of being engaged in management of gout. Themes are shown in the *bold gradient arrow* at the top of the figure, and the gradient represents the dynamic linkage among the themes. Beneath each theme are boxes containing the corresponding categories. *Solid black arrows* within each theme depict relationships between categories

The relationship between three themes (*interfering with management of gout* [theme 3], *processing the diagnosis and management of gout* [theme 1], and *supporting management of gout* [theme 2]) is presented in Fig. 1, which shows that becoming engaged in the management of gout is a dynamic process. It is important to note the position of the themes in the process of becoming engaged in the management of gout: *processing the diagnosis and management of gout* is at the center of the spectrum, mediating the transition between *interfering with management of gout* and *supporting management of gout*. Through processing the diagnosis and management of gout, participants gain an understanding of the causes of gout and discover methods by which to adapt to it. Within processing the diagnosis is *testing the waters*, which, based on the participants’ accounts, can move them toward either supporting or interfering with adherence to treatment. Furthermore, the categories *testing the waters* and *searching for reason*, located within the theme *processing the diagnosis and management of gout*, are connected, as demonstrated by a participant who mentioned, “*You’re trying to figure what are you doing, what are you intaking in your system,*” and the curiosity of dietary triggers caused the participant to “*test it for a while*” (*participant 2, male*). Intrinsic processes closely linked to participants *developing acceptance* are *seeing a difference* and understanding the *resonating importance of gout medications*. In noticing a change in their gout activity, many attributed that change to their medications, thereby reinforcing the importance of ULT. The combination of noticing an improvement in their health and taking ULT ultimately supported the development of acceptance in terms of actively managing their gout.

## Discussion

We conducted a qualitative study using a constructivist grounded theory approach to understand patients’ experiences with gout and how patients become engaged in the management of gout within the context of receiving care. Findings include one theme describing the experience of gout, specifically *processing the diagnosis and management of gout*, as well as perceptual and practical factors that influence the management of gout, which are distinguished as the themes *supporting management of gout* and *interfering with management of gout*. Furthermore, in exploring the relationships between study themes, we constructed an explanatory framework that explains how becoming engaged in gout management is a dynamic process whereby patients may transition through *interfering with management of gout* to *processing the diagnosis and management of gout* to *supporting management of gout*. As such, an implication of these findings is informing how healthcare providers can mediate this process to improve care delivery and health outcomes.

A considerable portion of prior qualitative research in gout has described barriers to adherence and management [16, 17, 30–42]; however, understanding of the factors that support optimal gout management is incomplete. We interviewed participants enrolled in a study of an eHealth-supported collaborative care intervention for gout, which gave us an opportunity to understand the determinants that support management of gout. Although direct elucidation of supporting factors is infrequent in prior literature, a review of available data revealed content related to three of our categories: *being organized* [16, 17, 31, 34, 41, 42], *identifying motivation* [16, 17, 31–33, 35, 42], and *taking control* [17, 32, 41]. Our present study contributes to the literature by constructing and comprehensively describing these categories.

A unique finding in this study was the integrated relationship among three categories—*developing acceptance*, *seeing a difference*, and *resonating importance of gout medications*—constructed within the theme *supporting management of gout*. These findings demonstrate the power of patient perceptions regarding illness and medications within the process of increasing engagement in the management of gout [43]. Moreover, this represents an opportunity for healthcare providers because they can encourage this resolution to develop acceptance by reviewing with patients their SUA over time, tracking gout activity, and discussing gout pathogenesis.

Along with a comprehensive description of elements that support gout management, key to our study is the development of an explanatory framework for conveying how patients with gout become engaged in managing their disease. Only two previous studies have described frameworks for understanding patients’ experiences with gout [17, 35]. In the first study, Richardson et al. reported determinants of ULT uptake and developed a framework describing ULT acceptance as dynamic, thus providing support for continual follow-up for patients with gout [35]. This study demonstrated findings similar to those in our study regarding how noticing a difference in gout symptoms can positively influence disease management [35]. The second study, by Singh et al., was focused on the experiences of African American male veterans with gout who were adherent to ULT and deductively conceptualized self-management using an existing framework, the Health Belief Model [17]. An advantage of the inductive framework constructed in our study is that findings are drawn directly from the patients’ perspectives and expand on the current literature regarding gout management to thereby impart healthcare providers with a basis for understanding the unique perceptions held by patients with gout. As patients enter the healthcare system, they hold beliefs that undoubtedly influence the impending course of management [27, 44], and as such, having healthcare providers attuned to these perceptual and practical factors along the continuum of gout management will inform opportunities to optimize care delivery. For example, when the behavior of taking control of gout management appears absent, healthcare providers can assist patients by providing a thorough plan for medication-taking and coping with gout flares, as well as encouraging patients to use recommended resources.

A unique feature of our qualitative study is that it is nested within an eHealth-supported collaborative care model for gout, which is well-suited to our aim of understanding how patients with gout can become engaged in managing their disease within the context of receiving care. In particular, this study adds to the comprehension of the patient experience with gout by constructing a theme to describe the *processing of the diagnosis and management of gout*. During the diagnosis, patients may search for reasons for having gout and the cause of gout flares, which is similar to a narrative described for U.K. patients with gout [32]. The behavior characterized as *testing the waters* in this study was predisposed by lacking knowledge about medications or being unconvinced of one’s susceptibility to future gout attacks. When participants modified their diet or ULT, often gout symptoms reappeared and would reinforce the need to be engaged in gout management. This process of receiving physiological feedback when *testing the waters* may be a feature unique to patients with gout, given that disease manifestations are fairly immediate. These findings emphasize the importance of providing continual follow-up beyond the initial diagnosis when patients may be inclined to trial medications or diet and allied healthcare providers are well-suited to supporting these key components of gout care.

There are strengths and limitations to this study that need to be considered. Strengths include the study design, because constructivist grounded theory uses techniques such as inductive analysis, constant comparison, and reflexivity to ensure that results are representative of the patient experience. Furthermore, we observed saturation in our study through simultaneous data gathering and analysis along with application of the constant comparative method. Limitations include the recruitment method, because individuals were perhaps inclined to discuss factors that support gout management, given their participation in a larger study evaluating a model of gout care and that those enrolled in research studies generally display healthier behaviors. The purposeful sampling strategy helped to compensate for this problem by selecting participants with both unmanaged and well-managed gout to represent a range of experiences. Another limitation is the recruitment being restricted to rheumatology practices, because the majority of individuals with gout are treated in primary care. However, detailed description allows the transferability of results, and the findings of this study are confirmed with qualitative publications derived from both rheumatology and primary practices.

## Conclusions

This study provides insight into factors that support optimal management and has constructed a framework for elucidating the process of becoming engaged in gout management. By understanding the entire continuum of patient engagement in gout management, healthcare providers, including rheumatologists as well as allied health professionals, can adapt care delivery to patients who require support in specific domains [45].
